# Biochar phosphorus concentration dictates mycorrhizal colonisation, plant growth and soil phosphorus cycling

**DOI:** 10.1038/s41598-019-41671-7

**Published:** 2019-03-25

**Authors:** Zakaria M. Solaiman, Lynette K. Abbott, Daniel V. Murphy

**Affiliations:** SoilsWest, UWA School of Agriculture and Environment, and the UWA Institute of Agriculture, The University of Western Australia, Perth, WA 6009 Australia

## Abstract

We aimed to determine the relationship between biochar properties and colonisation of roots by arbuscular mycorrhizal (AM) fungi in agricultural soil. We used a range of biochars that differed in pH, water holding capacity, C, N and P concentrations to investigate interactions between biochar and AM fungi. A glasshouse experiment was conducted with subterranean clover and wheat, amended separately with 34 sources of biochar (applied at 1% w/w), to investigate potential responses in a phosphorus (P) deficient agricultural soil. Plant growth responses to biochar ranged from positive to negative and were dependent on biochar P concentration, available soil P and AM root colonisation. The higher the nutrient P concentration in biochar, the lower was AM colonisation. Growth responses of wheat and clover to the application of various biochars were mostly positive, and their growth was correlated, but biochar contributions to soil fertility varied with biochar properties. When nutrient concentrations are higher in biochars, especially for P and N, plants can gain access to nutrients via the plant roots and mycorrhizal hyphae. Thus biochar amendments can increase both plant nutrient uptake and crop production in nutrient deficient soil.

## Introduction

Biochars are by-products of the pyrolysis process of burning biomass from either plant or animal origin heated (>250 °C) in a low or nil oxygen environment^1^. The carbon (C), ash and nutrient contents of biochars vary depending on the biomass source used as feedstock and the production temperature employed^2^. Biochars contain highly stable forms of C which have the potential to remain in the soil for hundreds of years^3–5^ but the ash only remains for a short time. Biochars are attracting attention as a stable soil amendment, as a C sink in agricultural soils, and as a stimulant of soil biological fertility^6^ and agricultural productivity^7–9^. Some biochars applied at high rates have been shown to increase soil water holding capacity either directly due to their high surface areas^10^ or indirectly in association with increases in soil organic carbon^11^. Biochars contain traces to high concentrations of nutrients including phosphorus (P)^8,12^. Plant responses to application of biochars to soil range from increased to the decreased colonisation of roots by arbuscular mycorrhizal (AM) fungi depending on P and nitrogen (N) concentrations in the biochars^13–17^. Biochar may also contain unwanted compounds such as crystalline silica, dioxin, phenolic compounds, volatile compounds and heavy metals based on sources^18–20^ which could influence mycorrhizal colonisation. Variability in the quality of biochars may lead to diverse effects on mycorrhizal colonisation, with the potential to enhance P uptake and growth of plants to different degrees, including positive responses under drought stress^21^.

There is an ongoing debate about the agronomic benefit of biochar on crop growth, soil fertility and AM symbioses. Where biochars have been shown to increase plant growth and yield^22,23^ and the involvement of AM fungi has been proposed, a range of possible mechanisms has been suggested^14,24^. Some studies reported an increase in mycorrhizal colonisation of roots in response to application of biochars without identifying the involvement of specific mechanisms^15,25–27^. It has been claimed that biochar can increase microbial activity, including that of mycorrhizal fungi, by providing a favourable microhabitat^24,25,27^. Extraradical hyphae of AM fungi can extend into biochar fragments buried in soil, sporulation can occur inside biochar pores^28^, and increased plant P uptake can result^13^. However, despite demonstrated influences of biochars on colonisation of roots by AM fungi, comparative effects of a wide range of biochar sources on AM colonisation have not been investigated.

Nevertheless, it is expected that the nature and extent of response to soil amendment with biochar will depend on the feedstock used to make the biochar and that this will be associated with variation in biochar physical characteristics such as pore sizes^24,29^, ash content and chemical characteristics^30^. Potential interactions between biochar and colonisation of roots by AM fungi could occur directly by altering the growth of hyphae in the soil prior to the colonisation of roots^17^, and indirectly by altering root growth and subsequent colonisation^15,17^. Biochar can also alter colonisation of roots by stimulating the growth of hyphae in the soil before the establishment of mycorrhizal symbiosis under water-limiting conditions^21^.

While biochar can influence colonisation in wheat roots by AM fungi both in the field and under glasshouse conditions^15,26^, a plant growth benefit gained from amending the soil with biochar can vary according to the properties of the biochar used^31^. Therefore, our study extended previous investigations by comparing 34 different biochars sourced from 17 biochar feedstocks. The aim was to determine the effects of biochar on plant growth and some aspects of soil fertility which could depend on interactions between biochar and indigenous AM fungi present in the agricultural soil used. It compared biochar effects on mycorrhizal colonisation, plant growth, and P nutrition of subterranean clover and wheat when applied to P-deficient agricultural soil. We tested the hypothesis that the higher P concentration in biochar will reduce mycorrhizal colonisation in roots of both subterranean clover and wheat by a naturally occurring community of AM fungi in the agricultural soil.

## Results

### Characteristics of biochars

The range of characteristics of the 34 different biochars (Table 1) demonstrated considerable variability, including their pH and water holding capacity (WHC). The pH of the biochars ranged from 3.7 to 12.3; most biochars were mild to strongly alkaline, and only two were acidic (B56 produced from green waste: pH 4.7 and B71 produced from woods and stored for long unknown periods: pH 3.7). The WHC of the biochars varied from 47% (for B70 made from oil mallee wood) to about 460% (for B43 made of from rice husk). The total C content of biochars varied from 19.29% (for B41 made from biosolids) to 85.43% (for B33 made from non-activated sawdust). The cases of low C are likely to be due to the presence of inorganic solids, including ash in the biochar. The total N concentration of the biochars varied from 0.06% (for B30 made from sawdust) to 2.56% (for B41 made from biosolids). Total P concentrations in the biochars ranged from <0.01% (for B32 made from sawdust) to 1.89% (for B54 made from poultry litter) and 2.83% (for B37 which was a biochar-mineral complex artificially formulated).

**Table 1:** Properties of biochars made from different feedstocks used in this experiment.

Biochar	Biochar name	Biochar feedstock	Production	pH	WHC*	Organic C	Total N	Total P
code			temp (°C)	(CaCl_2_)	(%)	%	%	%
B27	Simcoa	Jarrah	600	7.6	162	77.77	0.28	0.006
B29	Rice husks	Rice husks	600–700	8.4	247	35.93	0.29	0.115
B30	SD NA 450	Sawdust non-activated	450	4.6	257	59.33	0.06	0.002
B31	SD NA 550	Sawdust non-activated	550	12.1	277	83.49	0.17	0.014
B32	SD NA 600	Sawdust non-activated	600	6.6	317	70.99	0.11	0.003
B33	SD NA 750	Sawdust non-activated	750	11.5	257	85.43	0.27	0.008
B34	SD A 600	Sawdust activated	600	8.7	312	84.32	0.11	0.004
B35	SD A 700	Sawdust activated	700	11.4	257	83.95	0.11	0.013
B36	WC NA 550	Woodchip non-activated	550	7.4	187	83.51	0.11	0.004
B37	BMC (Anthroterra)	Mineral complex	400	7.4	72	32.02	1.06	2.830
B38	Green waste Homogenized	Green waste	—	7.7	232	67.63	0.09	0.011
B39	Paper mill	Petrie Mill Paper Sludge	500	7.5	112	32.19	0.36	0.393
B41	Ballina Biosolid	Biosolid	550	7.6	70	19.29	2.56	1.020
B42	Chicken manure	Chicken manure	550	7.6	110	35.28	1.60	1.250
B43	Rice Husk - BARMAC	Rice husk	—	9.5	462	31.55	0.29	0.152
B44	BEST Cow Manure	Cow manure	550	9.4	137	39.37	0.38	0.754
B53	Litter 1	Poultry	550	9.7	72	32.00	0.83	1.800
B54	Litter 2	Poultry	450	8.9	72	32.03	1.70	1.890
B55	Green waste 1	Green garden organics	550	7.6	187	75.60	0.16	0.016
B56	Green waste 2	Green garden organics	450	4.7	102	56.36	0.13	0.010
B57	Enhanced 1	GW 1 enhanced with N	550	8.3	112	72.99	0.35	0.030
B58	Enhanced 2	GW 1 enhanced minerals	450	9.2	132	56.54	0.49	0.991
B59	Agrichar GW	Green waste	500	9.2	175	70.91	0.62	0.173
B60	Agrichar WW	Wood waste	500	7.9	147	76.20	0.51	0.012
B61	RBE Macadamia Nut Shell	Macadamia Nut Shell	450–480	8.1	77	78.03	0.57	0.042
B63	RBE Wheat Stubble	Wheat trash	450–480	9.6	137	75.33	0.73	0.153
B64	AR-02	Oil Mallee (*E. polybratea*)	700	10.4	145	80.66	0.08	0.048
B65	AG-10	Oil Mallee (*E. polybratea*)	700	12.3	177	85.29	0.21	0.045
B66	AF-01	Oil Mallee (*E. polybratea*)	700	12.1	167	78.85	0.22	0.058
B67	CF-12	Oil Mallee (*E. polybratea*)	700	8.5	222	81.79	0.59	0.069
B68	CG-029	Oil Mallee (*E. polybratea*)	700	8.4	180	84.67	0.34	0.033
B69	CP-11	Oil Mallee (*E. polybratea*)	700	8.5	87	73.60	0.28	0.048
B70	CP-01	Oil Mallee (*E. polybratea*)	700	7.6	47	74.21	0.57	0.081
B71	Wundowie	Jarrah plus other woods	550–650	3.7	97	64.31	0.15	0.005

*WHC = water holding capacity.

### Biochar effects on colonisation of roots by AM fungi

**Figure 1:**
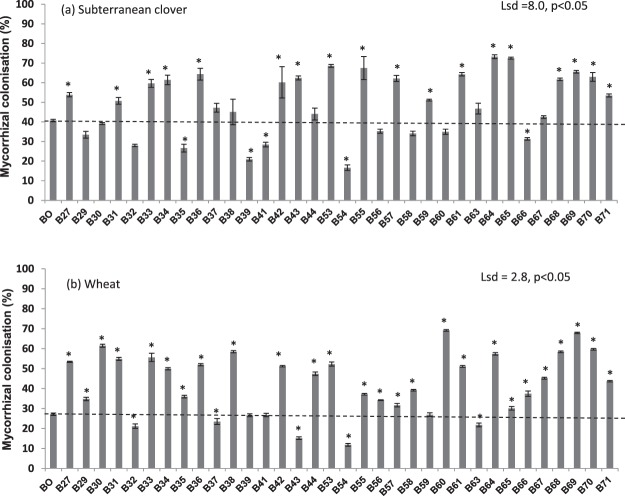
The effect of biochars on arbuscular mycorrhizal colonisation (%) in roots of (**a**) subterranean clover (lsd = 8.0, p < 0.05) and (**b**) wheat (lsd = 2.8, p < 0.05) after eight weeks of growth. Bar bigger than control (B0) with an asterisk (*) indicate increase but bar smaller than the control with an asterisk (*) indicate decrease. Bars do not have an asterisk do not differ.

Addition of the biochars to soil had variable effects, ranging from positive to negative, on the extent of colonisation of roots by AM fungi for both subterranean clover and wheat (p < 0.01; Fig. 1a,b). The highest level of mycorrhizal colonisation (%) of roots was observed for biochars B64 and B65 (both produced from oil mallee) in subterranean clover (Fig. 1a) and for biochars B69 (oil malle), B60 (wood waste) and B30 (sawdust) in wheat (Fig. 1b). The lowest mycorrhizal colonisation (%) was observed both in subterranean clover and wheat when poultry litter biochar B54 (biochar produced from poultry litter contains total P 1.89%, see Table 1) was applied. Overall, mycorrhizal colonisation (%) was generally higher in roots of subterranean clover than wheat across all biochars, and effects of biochars on root colonisation in these two test plant species were correlated (r = 0.40, p < 0.05).

### Biochar effects on plant growth and P nutrition

**Figure 2:**
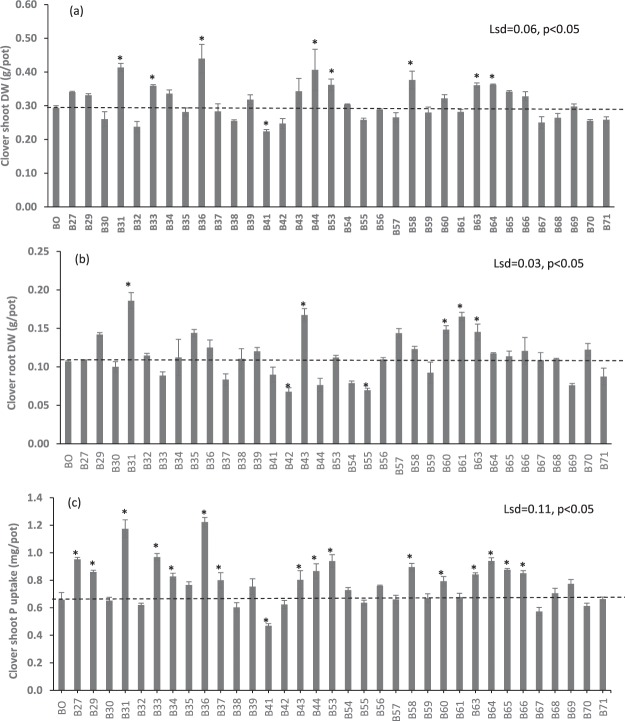
The effect of biochars on (**a**) shoot DW, (**b**) root DW, and (**c**) P uptake of subterranean clover after eight weeks of growth. Bar bigger than control (B0) with an asterisk (*) indicate increase but bar smaller than the control with an asterisk (*) indicate decrease. Bars do not have an asterisk do not differ.

**Figure 3:**
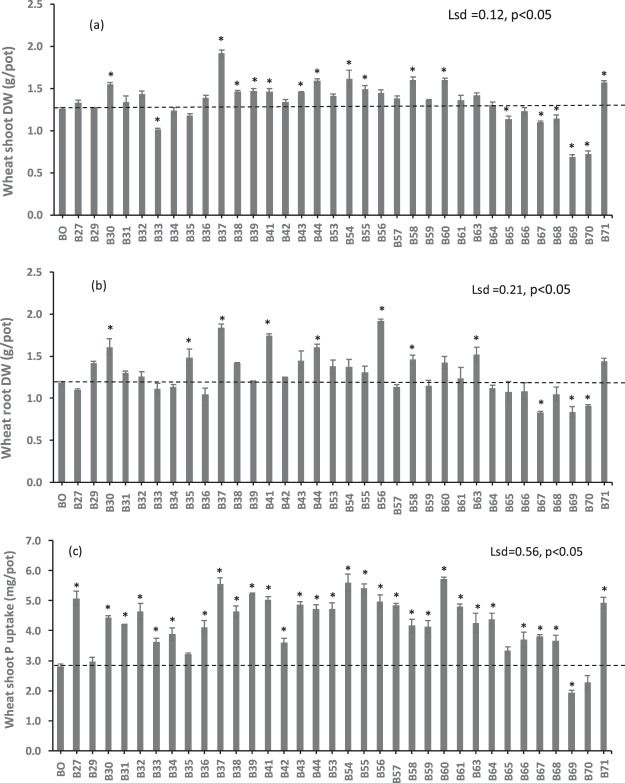
The effect of biochars on (**a**) shoot DW, (**b**) root DW, and (**c**) P uptake of wheat after eight weeks of growth. Bar bigger than control (B0) with an asterisk (*) indicate increase but bar smaller than the control with an asterisk (*) indicate decrease. Bars do not have an asterisk do not differ.

Shoot and root growth of subterranean clover and wheat responded to biochar application as either increases or decreases depending on the sources of biochar (p < 0.05; Figs 2 and 3). In subterranean clover, eight biochars significantly increased shoot DW, and it was decreased by one biochar (Fig. 2) whereas root DW was increased by five biochars and decreased by two biochars. Similarly, P uptake in subterranean clover was increased by 16 biochars and decreased by only one biochar (p < 0.05; Fig. 2). In wheat, 12 biochars significantly increased shoot DW, and it was decreased by six biochars (Fig. 3) whereas root DW was increased by eight biochars and decreased by three biochars. For P uptake in wheat, increases were observed for 29 biochars and a decrease for only one biochar (p < 0.05; Fig. 3).

### Biochar effects on soil P availability at the end of plant growth cycle

**Figure 4:**
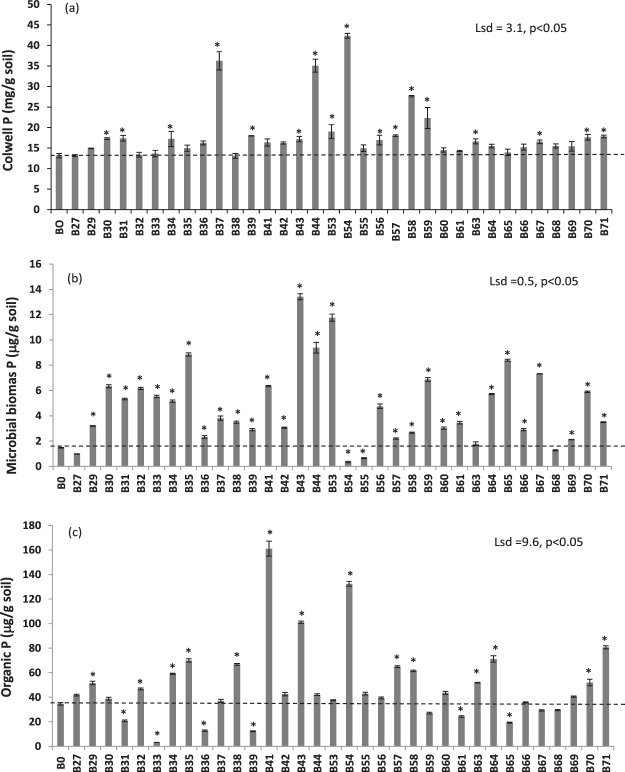
The effect of biochars sources on (**a**) inorganic Colwell P (lsd = 3.1, p < 0.05), (**b**) microbial biomass P (lsd = 0.5, p < 0.05) and (**c**) organic P (lsd = 9.6, p < 0.05) in soil after 8 weeks of growth. Bar bigger than control (B0) with an asterisk (*) indicate increase but bar smaller than the control with an asterisk (*) indicate decrease. Bars do not have an asterisk do not differ.

Soil P fractions after harvest were significantly influenced by biochar source (Fig. 4). The highest level of residual soil P at the time of plants was harvested observed for soil amended with biochars B37 (biochar-mineral complex), B44 (cow manure) and B54 (poultry litter). The second highest residual soil P was recorded for soil amended with biochars B58 (green waste enhanced with N) and B59 (green waste). Similar amounts of available P remained in soil amended with most of the other biochars at the end of plant growth cycle. Microbial biomass P was lowest in soils amended with biochars B27 (jarrah wood), B54 (poultry litter), B55 (green garden organics) and B68 (oil mallee) following eight weeks of plant growth. Soil organic P concentration was highest in soil amended with biochar B41 (biosolid) and lowest in soil amended with biochar B33 (sawdust) following eight weeks of plant growth.

### Correlations between soil or biochar properties and plant parameters

Significant Pearson’s correlation coefficients (*r*) were observed between extractable soil P and all measured parameters of plant growth and % mycorrhizal colonisation (Table 2). Soil NaHCO_3_-extractable P (Colwell P) was correlated with wheat shoot biomass, uptake of P in wheat plants (Table 2), with biochar total P (r = 0.74*, P < 0.05), and with biochar total N (r = 0.39*, P < 0.05).

**Table 2:** Pearson’s correlation coefficients (r) between the soil or biochar properties, measured parameters of plants and % mycorrhizal colonisation after 8 weeks of growth.

Measured parameter	Mycorrhizal colonisation		Shoot dry weight		Shoot P uptake	
	Subterranean clover	Wheat	Subterranean clover	Wheat	Subterranean clover	Wheat
Soil pH	−0.05	−0.06	0.14	−0.14	0.08	−0.04
Soil EC	−0.01	−0.12	−0.12	0.080	−0.15	0.01
Soil available P	−0.30**	−0.35***	0.16	0.50***	0.05	0.35***
Microbial biomass P	0.14	−0.08	0.13	−0.05	0.05	−0.05
Soil organic P	−0.30**	−0.37***	−0.30**	0.25*	−0.39***	0.27**
Biochar pH	0.15	−0.06	0.50***	−0.32***	0.46***	−0.33***
Biochar EC	−0.11	−0.37***	−0.26**	0.26**	0.01	−0.04
Biochar WHC	0.01	−0.10	0.16	−0.05	0.18*	−0.09
Biochar C	0.34***	0.39***	0.12	−0.48***	0.22*	−0.40***
Biochar N	−0.25**	−0.31**	−0.30**	0.21*	−0.31**	0.21*
Biochar P	−0.37***	−0.33***	−0.01	0.50***	−0.03	0.33***
Biochar K	−0.14	−0.34***	0.21*	0.31**	0.02	0.08

NS: not significant; *Significant at *p* ≤ 0.05; **Significant at *p* ≤ 0.01; ***Significant at *p* ≤ 0.001.

**Figure 5:**
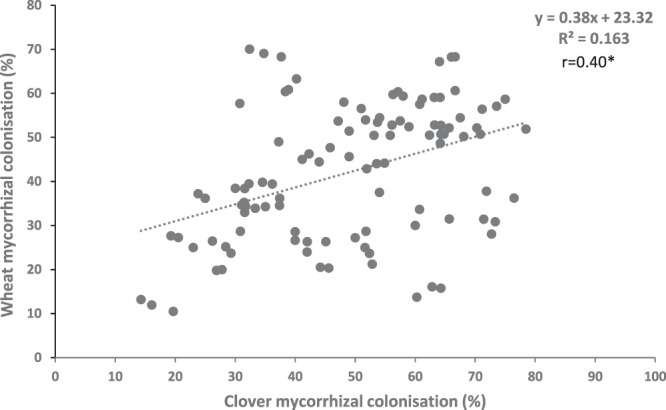
Correlation between subterranean clover mycorrhizal colonisation (%) vs wheat mycorrhizal colonisation (%) shown in scatter plot.

Significant Pearson’s correlation coefficients (*r*) were also observed between the soil pH and concentration of P in plant shoot tissues (Table 2). The availability of P in soil and % mycorrhizal colonisation were negatively correlated (Table 2). Soil microbial P was not significantly correlated with % mycorrhizal colonisation, plant growth or P uptake (P > 0.05), but organic P concentration in soil after plant growth was significantly and negatively correlated with % mycorrhizal colonisation in both plants (P < 0.05). Soil organic P concentration was positively correlated with plant growth and P uptake for wheat but negatively correlated for subterranean clover (P < 0.05). Biochar P concentration was negatively correlated with % mycorrhizal colonisation but positively correlated with plant growth and P uptake in wheat but not for subterranean clover (P < 0.05). Biochar N and K concentrations were both negatively correlated with % mycorrhizal colonisation (P < 0.05). Across all biochars, there was a significant correlation between % mycorrhizal colonisation and plant growth (p < 0.05) and with P uptake (p < 0.05) for subterranean clover. In contrast, there was no significant correlation between % mycorrhizal colonisation and plant growth for wheat (p > 0.05). There was a no significant correlation between shoot biomass of subterranean clover and wheat (p > 0.05) or between root biomass of subterranean clover and wheat (p > 0.05). However, there was a positive correlation between % mycorrhizal colonisation of subterranean clover and wheat (p < 0.05; Fig. 5).

**Figure 6:**
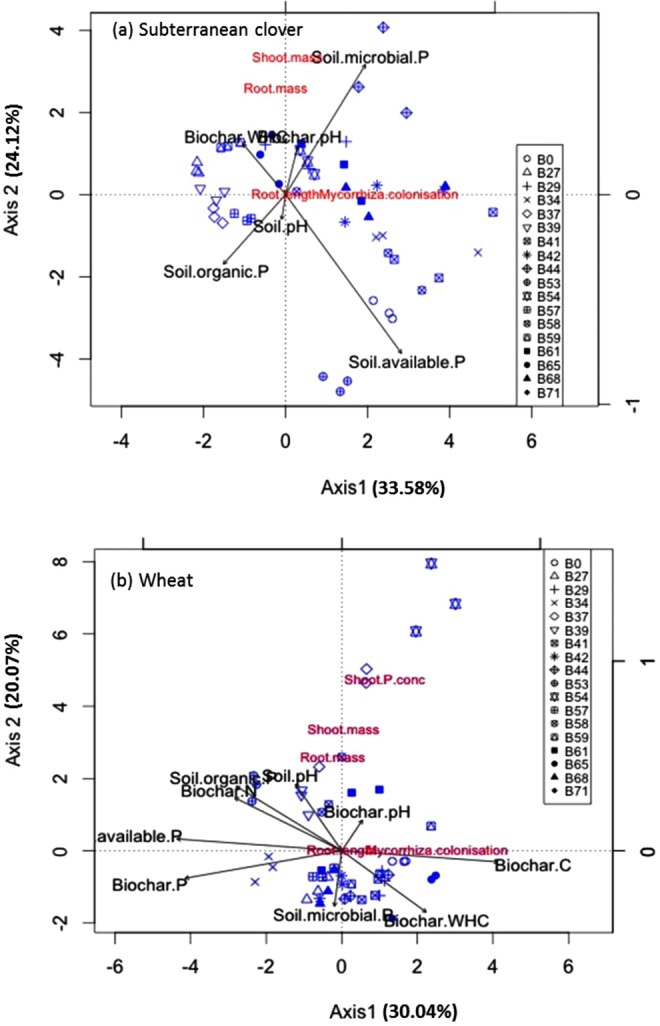
Principal component analysis ordination diagram Axis 1 (PC 1) vs. Axis 2 (PC 2) of (**a**) subterranean clover and (**b**) wheat parameters (mycorrhizal colonisation, shoot and root mass, P concentration in the shoots) for samples of the 17 sources of feedstocks plus a control. The percentage of total variance as explained by each axis is shown.

Principal component analysis (PCA) revealed patterns in plant parameters across all samples (Fig. 6a,b). For subterranean clover, PCA axis 1 was mostly influenced by soil organic and inorganic P and soil pH, whereas PCA axis 2 was most influenced by biochar pH and biochar WHC (Fig. 6a). However, the reverse occurred for wheat (Fig. 6b). The scatterplot showed slightly overlapping groups of samples corresponding to particular biochar types. The sample differentiation pattern concerning plant parameters was pronounced regarding biochar sources. The most symptomatic differences were observed for subterranean clover among biochar types. Increased % mycorrhizal colonisation in subterranean clover corresponded to enhanced P concentration with both shoots and roots, whereas wheat was characterised by lower % mycorrhizal colonisation and increased microbial biomass P.

## Discussion

Biochars prepared from different feedstock sources can differ in their influence^14^ including their effects on colonisation of roots by AM fungi and P availability, but the mechanisms involved are poorly understood. It was expected that biochars of different feedstock sources would differ in the extent to which they influenced root growth and that this would be associated with differences in nutrient concentrations in the biochar and the plant species used. It has been observed previously that P and N, in particular, may become more available to plants after soil is amended with some forms of biochar^8,32^ and this could increase root growth and % mycorrhizal colonisation. Increased root growth could also reduce the proportion of roots colonised by AM fungi, but increase the volume of roots colonised^21^.

Effect of biochars on mycorrhizal colonisation, plant growth and P nutrition have been reported previously for a small number of biochars using both wheat and subterranean clover^15,33^. In the present experiment, where we compared a much wider range of biochar sources, the higher the nutrient (P and N) concentrations in biochar, the lower was mycorrhizal colonisation and vice versa. Mycorrhizal colonisation (assessed as % of root length colonised) decreased with excess available P derived from biochars and soil^32,34,35^. Our experiment provides further support for the role of the mycorrhizal symbiosis in soil fertility and plant P nutrition when some sources of biochar are applied^36,37^. This needs further evaluation in other soil types and environmental conditions. Previous studies showed biochars had relatively small amounts of nutrients available to plants, which favoured colonisation of roots by microbes (including AM fungi)^23,38,39^. P-rich biochars could function as slow-release fertiliser^40^, thus available P is a factor that needs to be considered when selecting biochars for use as a soil amendment; an increase in available P in soil was observed in our experiment for many biochars.

Soil amendment with biochars can change soil physicochemical properties which may lead to increases in soil pH and nutrient availability and subsequent alterations in root colonisation by mycorrhizal fungi^13,23^. In our experiment, correlations between mycorrhizal colonisation and plant growth as well as between mycorrhizal colonisation and P uptake were observed in subterranean clover but not in wheat. This is likely to be because wheat is less responsive to mycorrhizas than subterranean clover^41^. Plant P uptake has been shown to increase with increasing level of biochar application in some agricultural ecosystems^7,33^. It is possible that biochar applications up to a certain level may stimulate mycorrhizal colonisation, leading to increased P uptake, but when applied at higher levels that enrich soil P beyond that required to overcome a deficiency for plant growth, the response may disappear^32^.

The physical and chemical properties of biochars (see Table 1) used in this experiment varied widely because the biochars were made from various feedstock sources and pyrolysis conditions^42^. Some biochar has the potential to provide a habitat for soil microbes, but this capability depends on the physical properties of biochar such as porosity and surface area^24^. Soil solution, air and water diffuse through the biochar pores facilitating soil microbes to colonise the biochar^24,43,44^. However, these claimed mycorrhizal interactions with biochars^14,27^ have only been investigated for a relatively small number of biochars^24^. For example, woody biochar from *Pinus radiata* increased fungal and bacterial abundance and supported a higher abundance of P solubilising bacteria^45^. Fungi, especially saprotrophic fungi, were shown to colonised biochar particles in association with decomposing fibrous organic matter^46^. Furthermore, any stimulation of colonisation of roots by AM fungi may also depend on soil characteristics^29^ and soil water availability^21^.

Although most biochar amendments used in our experiment increased mycorrhizal colonisation, plant growth and P uptake under glasshouse conditions in plastic lined confined pots, the scarcity of published data restricts evaluations of the potential of biochars produced from various sources for use as amendments under field conditions. Furthermore, the influence of biochars on mycorrhizal colonisation and effects on plant growth and P uptake under mixed cropping systems varied with biochar source and the reasons for this may be complex. This is the first report that demonstrates, for a large number of biochars with a wide range of characteristics, biochar effects on colonisation of roots by AM fungi. However, where biochars are produced as a soil amendment, appropriate application levels^32^ and the mechanisms underlying their effectiveness need to be investigated before field application is widely recommended^47^. Further studies on the effect of biochars which have a high potential as a habitat for mycorrhizal fungi are also necessary to assess bio-physicochemical interactions between biochar particles, soil, microbes and roots^48^.

As most of the biochars used here were prepared from diverse feedstocks that contain varying amounts of C and ash (which is unknown) they are likely to be extremely heterogeneous and impure when mixed with soil^49^. The ash that arises during the anaerobic burning process (pyrolysis) contains calcium carbonates (hence pH effects), struvite and calcium phosphate, and sometimes silicates, for example, in rice husks biochar (hence fertiliser effects). Conclusions about biochar efficacy are often based on investigations of only one to a few sources of biochar. However, the wide range of responses observed in our study of biochars from 17 different feedstocks (including multiple sources from similar feedstocks), highlights the need for caution when generalising about the effects of biochar. As such, we note conspicuous differences in biochar properties (e.g. the low pH in B32 and the differences between B55 and B56), suggesting that biochar’s final properties cannot be easily predicted from the information provided about the specific charring process. We demonstrated substantial differences in the efficacy of different sources of biochar in relation to the colonisation of roots by AM fungi. The possibility of sourcing large numbers of biochars produced under equivalent conditions is very unlikely and extends likely differences in biochar responses, even from the same feedstock. The conditions under which biochars are produced further compounds variability in efficacy. Therefore, in order to evaluate the potential benefits of biochars for use in the field, investigations need to focus on the responses of forms of biochars that are available locally.

## Methods

### Experimental design

Wheat (*Triticum aestivum* L. var. ‘Wyalkatchem’) and subterranean clover (*Trifolium subterraneum* L. var. ‘Seaton Park’) were grown together for 8 weeks under glasshouse conditions in an agricultural soil collected from Mingenew, Western Australia following its amendment with one of 34 biochars plus one control where no biochar was applied to the soil. Biochar codes (B27 and so on in Table 1) were used according to the Australian National Project Biochar Database (funded by the Department of Agriculture, Australian Government). There were three replicates of each treatment.

Each pot contained 1.4 kg air-dry soil which had been mixed thoroughly with one of the 35 biochar treatments at the rate of 1% (w/w). The 1.3 L plastic pots were sown with seeds of wheat and subterranean clover and thinned to 2 plants of each species per pot after germination. Each pot was watered to 70% of field capacity based on the daily addition of water to the prescribed weight.

### Soil and biochar characteristics

Soil (0–10 cm) was collected from a subterranean clover/wheat rotation near Mingenew, Western Australia (latitude 29°19′, longitude 115°44′). Mingenew has a Mediterranean climate and a mean annual rainfall of 400 mm (with 80% falling in the May to October growing season). The soil contained 85% sand, 3% silt and 12% clay and was classified as a Tenosol (sand over gravel)^50^ and Humic Dystroxerepts^51^; the 0–10 cm layer was analysed for basic properties. The pH of the soil was 4.8 measured in 0.01 M CaCl_2_ at 1:5 (w/v) ratio. Organic matter was 10.3 g/kg soil measured by dry combustion using an elementar (vario MACRO CNS; Elementar, Germany). The soil contained 0.6 g/kg total N, 7 and 5 mg/kg NO_3_-N and NH_4_-N respectively. This soil was chosen because its loamy sand texture gave it a low capacity to retain P and the low available P (7.5 mg/kg) content was suitable for a mycorrhizal response in both subterranean clover and wheat. Soil P and K were measured using the 0.5 M NaHCO_3_ extraction method^52^. Soil available P, organic P and microbial biomass P were extracted after harvest of plants^52–54^. The P concentration in the extracts was measured in a spectrophotometer^55^.

The 35 biochars including one control (no biochar amended) used in this evaluation were produced from 17 sources of feedstocks (see Table 1) manufactured under different temperature conditions and sieved to 2 mm. The pH of the biochars was measured in 0.01 M CaCl_2_ at 1:5 (w/v) ratios. Water holding capacity of the biochars was measured using a gravimetric method^56^. A subsample of biochar was finely ground before total C and N contents were determined by dry combustion using an elementar (vario MACRO CNS; Elementar, Germany). Total P in biochars was measured after digested in 3:1 HNO_3_-HClO_4_ and P measured in solution by the molybdenum-blue method^55^.

### Assessment of colonisation by AM fungi

At harvest after washing with tap water, sub-samples of roots (0.5 g) were cut into approximately 1 cm pieces and cleared in 10% KOH, acidified and stained with Trypan blue (0.05%) in lactoglycerol (1: 1: 1.2/ lactic acid: glycerol: water)^57^. Mycorrhizal colonisation (%) of roots by AM fungi was assessed using the gridline root intercept method under a microscope at 100 × magnification as % root colonised^57^.

### Plant analyses

At harvest, shoots were cut from each plant and roots were washed free of soil and organic matter. Shoots and roots (after a defined weight of roots was removed for assessment of AM colonisation) were dried at 60 °C for at least 72 h to determine the shoot and root dry weights (DW). Oven-dried shoots were ground and digested in 3:1 HNO_3_-HClO_4_ mixture and the P concentration in the digest measured by the molybdenum-blue method^55^. Shoot P uptake was calculated by multiplying shoot P concentration by shoot weight.

### Statistical Analyses

Statistical analyses were carried out using Genstat (v.18). One-way analysis of variance was used to detect significant effects of biochars on all soil and plant parameters measured. The least significant difference (LSD) was applied to test significance between means. The significant Pearson’s correlations between the measured soil and plant parameters after eight weeks of wheat and subterranean clover growth were tested. Plant parameters (shoot and root mass, P concentration in shoots) and mycorrhizal colonisation were also explored with principal component analysis (PCA) to identify the association between these traits and to recognise the grouping of samples, associated with the soil properties and 17 different category based on biochar sources, with their similar source and characteristics. The analysis was based on the correlation matrix and multivariate analyses performed for each plant species separately.
